# Thymoquinone challenges UHRF1 to commit auto-ubiquitination: a key event for apoptosis induction in cancer cells

**DOI:** 10.18632/oncotarget.25583

**Published:** 2018-06-19

**Authors:** Abdulkhaleg Ibrahim, Mahmoud Alhosin, Christophe Papin, Khalid Ouararhni, Ziad Omran, Mazin A. Zamzami, Abdulrahman Labeed Al-Malki, Hani Choudhry, Yves Mély, Ali Hamiche, Marc Mousli, Christian Bronner

**Affiliations:** ^1^ Institut De Génétique Et De Biologie Moléculaire Et Cellulaire (IGBMC), INSERM U1258 CNRS UMR 7104, Université de Strasbourg, Illkirch, France; ^2^ BioTechnology Research Center (BTRC), Tripoli, Lybia; ^3^ Department of Biochemistry, Cancer Metabolism and Epigenetic Unit, Faculty of Science, King Abdulaziz University, Jeddah, Saudi Arabia; ^4^ Cancer and Mutagenesis Unit, King Fahd Medical Research Center, King Abdulaziz University, Jeddah, Saudi Arabia; ^5^ College of Pharmacy, Umm Al-Qura University, Makkah, Saudi Arabia; ^6^ CNRS UMR 7021 Laboratoire de Bioimagerie et Pathologies, Université de Strasbourg, Faculté de Pharmacie, Illkirch, France

**Keywords:** apoptosis, thymoquinone, tumor suppressor genes, ubiquitination, UHRF1

## Abstract

Down-regulation of UHRF1 (Ubiquitin-like containing PHD and Ring Finger 1) in Jurkat cells, induced by natural anticancer compounds such as thymoquinone, allows re-expression of tumor suppressor genes such as *p73* and *p16*^*INK4A*^. In order to decipher the mechanisms of UHRF1 down-regulation, we investigated the kinetic of expression of HAUSP (herpes virus-associated ubiquitin-specific protease), UHRF1, cleaved caspase-3 and p73 in Jurkat cells treated with thymoquinone. We found that thymoquinone induced degradation of UHRF1, correlated with a sharp decrease in HAUSP and an increase in cleaved caspase-3 and p73. UHRF1 concomitantly underwent a rapid ubiquitination in response to thymoquinone and this effect was not observed in the cells expressing mutant UHRF1 RING domain, suggesting that UHRF1 commits an auto-ubiquitination through its RING domain in response to thymoquinone treatment. Exposure of cells to Z-DEVD, an inhibitor of caspase-3 markedly reduced the thymoquinone-induced down-regulation of UHRF1, while proteosomal inhibitor MG132 had no such effect. The present findings indicate that thymoquinone induces in cancer cells a fast UHRF1 auto-ubiquitination through its RING domain associated with HAUSP down-regulation. They further suggest that thymoquinone-induced UHRF1 auto-ubiquitination followed by its degradation is a key event in inducing apoptosis through a proteasome-independent mechanism.

## INTRODUCTION

Inactivation of tumor suppressor genes (TSGs) is a common characteristic in human cancer cells. Mutations, ubiquitination-dependent degradation and epigenetic silencing are the main mechanisms involved in the regulation of TSGs [1–4]. Epigenetic silencing of TSGs is mainly operated through DNA methylation [5]. Of note, the sole down-regulation of UHRF1 (Ubiquitin-like containing PHD and Ring Finger 1) is sufficient to allow re-expression of several TSGs including, *RB1*, *p16*^INK4A^*, KISS1, BRCA1, RASSF1*, *CDKN2A* and *RARα* [6], meaning that all the epigenetic marks read or catalysed by UHRF1 are involved and not more. These epigenetic marks are DNA methylation, H3K9me2/3, H3R2 and putatively histone ubiquitination [7–16]. Thus, UHRF1 can be considered as a master regulator of TSGs as it coordinates DNA methylation and histone modifications at their promoters [13, 14, 17–19].

UHRF1, a potent oncogene overexpressed in many human cancer cells, plays an important role in G1/S transition and the epigenetic silencing of various TSGs such as *p16*^INK4A^, *p14*^ARF^, *BRCA1* and *RB1* [6–8, 20–27]. UHRF1 down-regulation induces reactivation of TSGs and apoptosis in cancer cells [22–24, 28, 29]. UHRF1 is a member of a macromolecular complex including DNMT1, HDAC1, G9a, Tip60, RB1 and histone H3 [7, 20, 25, 30–33]. By its original structure, UHRF1 could be the “driver” of this complex to duplicate the epigenetic code after DNA replication and allows cancer cells to maintain gene repression, and in particular that of TSGs [7, 25].

E3 ligases, among which UHRF1, mediate the attachment of several ubiquitin molecules, termed polyubiquitination, to target proteins, thereby regulating protein degradation, cell cycle progression, DNA repair and transcription. E3 ligases can also catalyze the attachment of a single molecule of ubiquitin molecule, termed mono-ubiquitination. UHRF1 can catalyze both, polyubiquitination and monoubiquitination that have distinct and quite opposite roles. Histone ubiquitination has an important role in the regulation of chromatin structure and gene transcription. In this context, it has been demonstrated that mouse UHRF1 (Np95), via its RING domain, has specific E3 ubiquitin ligase activity for histone 3 [34]. More recently, the relevance of histone H3 ubiquitination by UHRF1 has been deciphered [11]. Indeed, UHRF1 ubiquitinates H3K23, which is a signal for the recruitment of DNMT1 to the replication fork and thus couples maintenance DNA methylation and replication [11, 14].

Natural drugs exhibiting anti-cancer properties have in common the ability to allow the re-expression of TSGs [7], but the mechanism involved remains a mystery. Among, these natural compounds, thymoquinone (TQ), which is the bioactive compound of the volatile oil derived from seeds of *Nigella sativa* plant, has potent selective anti-proliferative and pro-apoptotic properties towards a wide range of cancer cells versus normal cells [7, 29, 35]. In our previous study, we have shown that TQ inhibits cell proliferation and induces apoptosis in the p53-deficient *acute lymphoblastic leukemia* cell line (Jurkat cells) and this effect is associated with UHRF1 down-regulation and p73 up-regulation [29]. Recently, it has been shown that Shikonin, a natural naphthoquinone isolated from the Chinese traditional medicine Zi Cao (purple gromwell) involves the same pathway [36]. Of note, we have shown that conversely, UHRF1 is also able to decrease p73 expression [37].

We postulated that the overexpression of UHRF1 observed in cancer cells could be a result of an alteration of the degradation pathways, pointing out the interest of investigating the degradation pathways of UHRF1, which is one of the goals of the present study. It has been shown that HAUSP (herpes virus-associated ubiquitin-specific protease), also known as Ubiquitin Specific Protease 7, is found in the same complex as UHRF1 and DNMT1 to deubiquitinate and to protect them from degradation by the proteasome [18, 38, 39]. Indeed, HAUSP down-regulation induces UHRF1 and DNMT1 ubiquitination leading to their degradation via a proteasome-dependent process [18] but the downstream events remain to be deciphered.

The aim of the present study was to understand the mechanisms by which TQ can induce UHRF1 down-regulation and to determine the molecular events associated with such effect. Our results showed that TQ induces a rapid UHRF1 ubiquitination associated with HAUSP down-regulation followed by p73 up-regulation in Jurkat cells and HeLa cells. Point mutation of the RING finger of UHRF1 abrogates ubiquitination of UHRF1 induced by TQ, indicating that UHRF1 commits an auto-ubiquitination through its RING finger domain in response to TQ. Taken together, our results showed that TQ selectively induced a rapid UHRF1 auto-ubiquitination in cancer cells, which could be a result of HAUSP down-regulation.

## RESULTS

### TQ induces apoptosis and UHRF1 down-regulation

**Figure 1:**
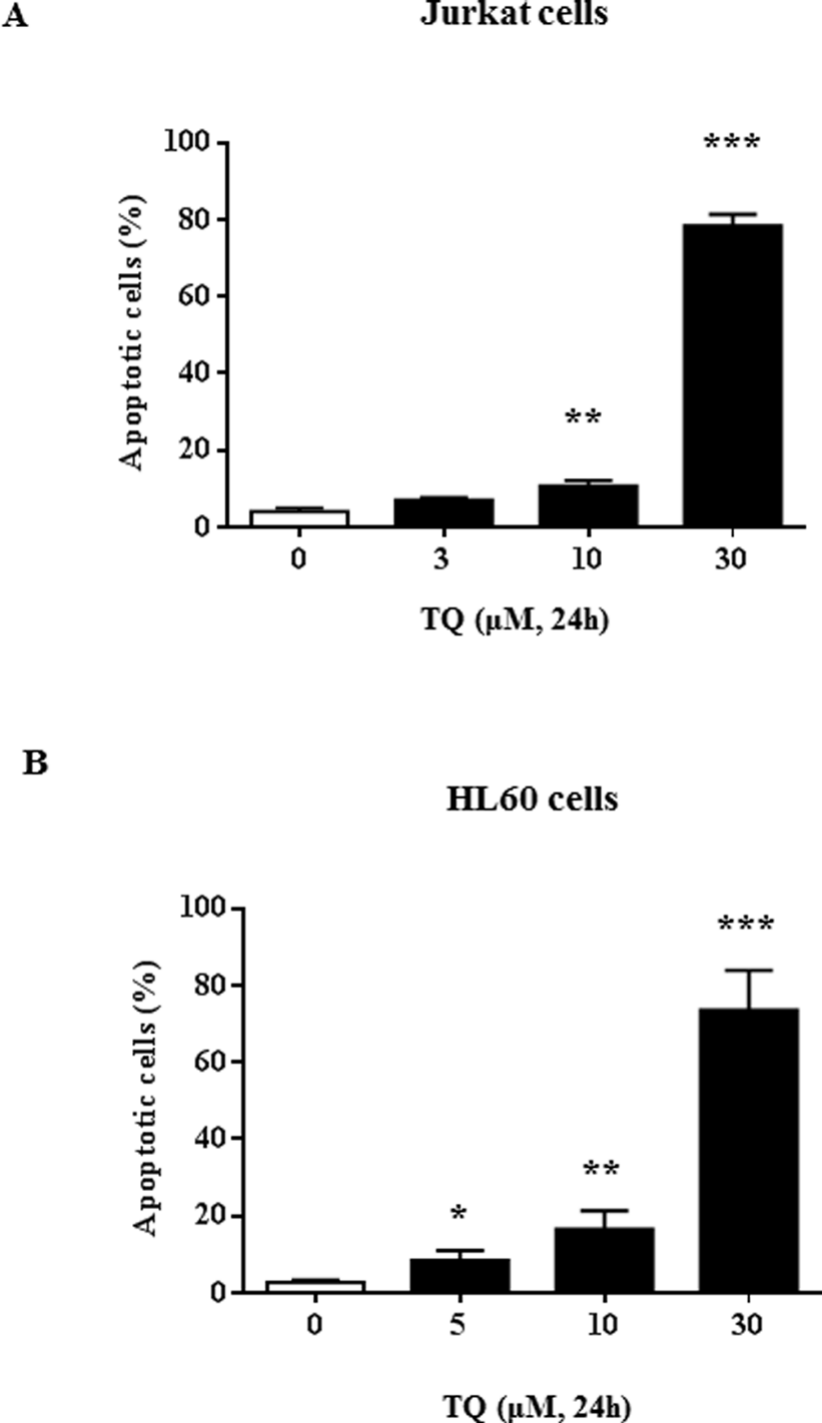
TQ-induced UHRF1 apoptosis in Jurkat and HL60 cells Jurkat cells (**A**) or HL60 cells (**B**) were exposed to increasing concentrations of TQ for 24 h. Apoptosis was assessed by flow cytometry using the Annexin V/7AAD staining apoptosis assay as described in the Materials and Methods section. Values are shown as means ± S.E.M. (*n* = 3); ^*^p < 0.05, ^**^p < 0.01, ^***^p < 0.001 versus control.

**Figure 2:**
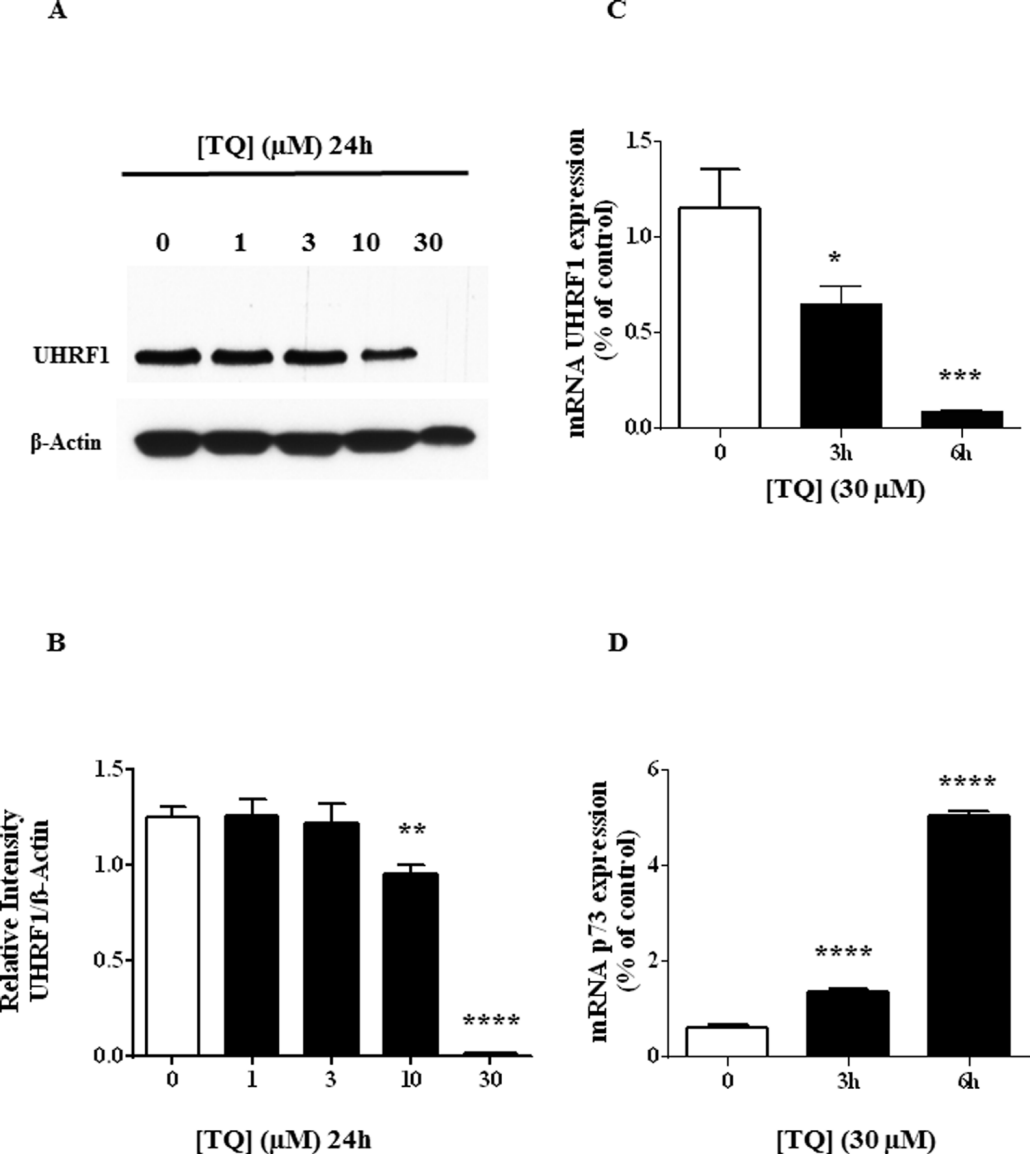
Effect of TQ on UHRF1 protein, UHRF1 mRNA and p73 mRNA (**A**) Expression of UHRF1 was analyzed by western blot after a 24 hr-treatment of Jurkat cells with various doses of TQ. (**B**) Quantification of UHRF1 expression using NIH ImageJ software. (**C** and **D**) *UHRF1* and *p73* gene transcription was investigated in Jurkat cells treated with 30 µM for the indicated times and the expression of both genes was investigated using RT-PCR as described in Materials and Methods. Values are shown as means ± S.E.M. (*n* = 3); ^**^p < 0.01, ^***^p < 0.001, ^****^p < 0.0001 versus control.

We have previously observed that TQ induced a dose-dependent down-regulation of UHRF1 in Jurkat cells [29] but the mechanism remained to be deciphered. Here, we confirmed that 30 µM of TQ induced apoptosis of about 80% of the Jurkat cells (Figure 1A) and of HL60 cells (Figure 1B). This concentration of TQ led to a complete disappearance of UHRF1 in Jurkat cells (Figure 2A). Quantification of UHRF1 protein expression showed that the inhibition effect significantly started from 10 µM (Figure 2B). Interestingly, we could observe a time-dependent disappearance of *UHRF1* mRNA that was completed after 6 hrs (Figure 2C) and that was inversely correlated with *p73* mRNA (Figure 2D). This result suggests that the down-regulation of UHRF1 eliminates the repression exerted on *p73* expression.

### TQ induces an early UHRF1 ubiquitination in jurkat cells

**Figure 3:**
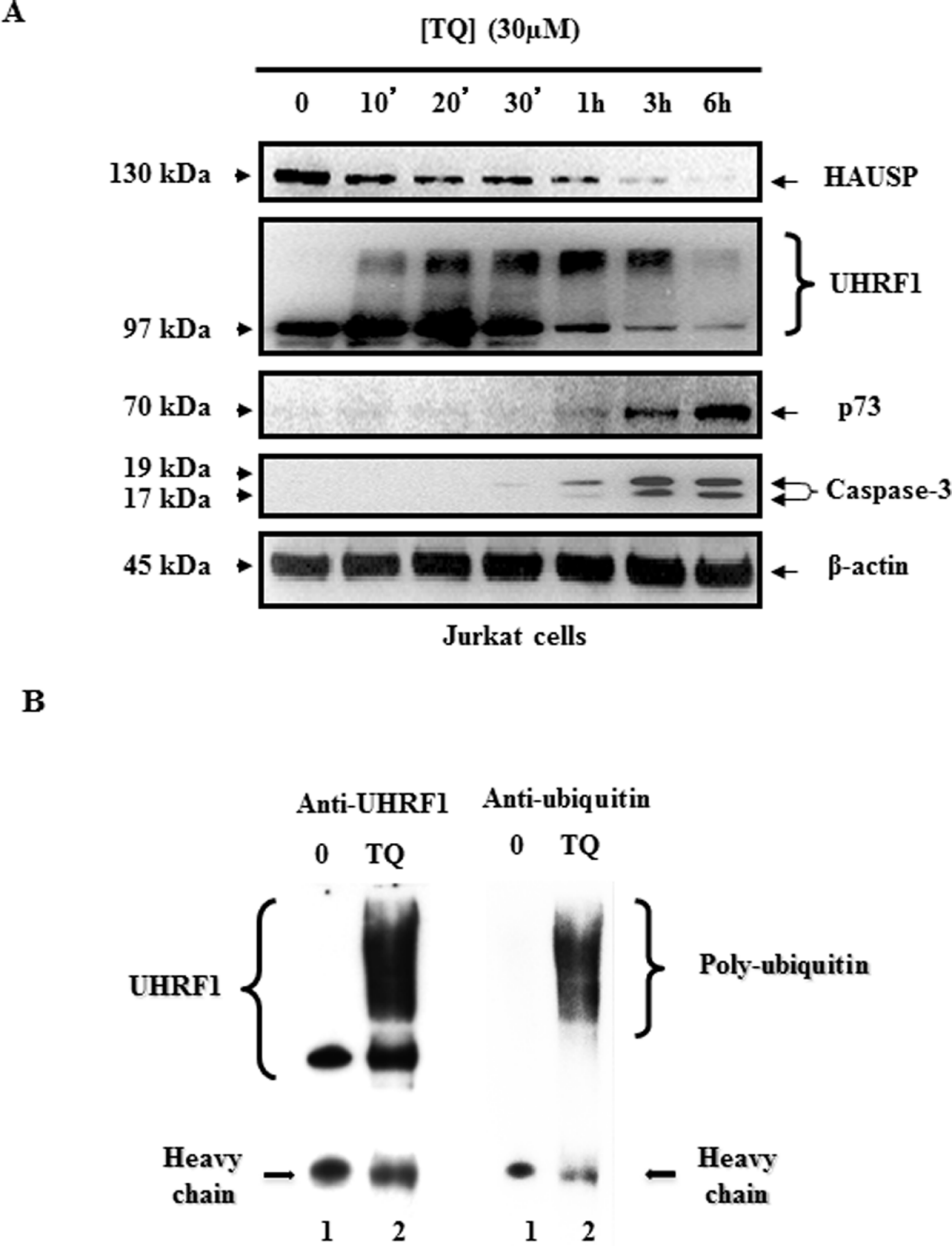
Time course effect of TQ on HAUSP, UHRF1, ubiquitinated UHRF1, p73 and cleaved caspase- 3 (**A**) Time course effect of 30 µM TQ on HAUSP, UHRF1, p73 and cleaved caspase- 3 expression in Jurkat cells. (**B**) Effect of TQ on UHRF1 ubiquitination in Jurkat cells. Cells were treated with 30 µM of TQ for 10 minutes. UHRF1 was immunoprecipitated from cell lysates as described in material and methods, then WB was performed using anti-UHRF1 antibody (lanes 1 and 2) or anti-ubiquitin antibody (lanes 3 and 4). Data are representative of 3 different experiments.

As a first step, we performed a kinetic analysis of TQ on UHRF1 expression in Jurkat cells. Time-course effects of 30 µM TQ on UHRF1 expression in Jurkat cells showed that UHRF1 began to decrease after 1 hr and disappeared almost completely after 6 hrs of treatment (Figure 3A). A closer analysis revealed that higher bands than the usual one at 97 kDa appeared already after 10 min (Figure 3A). Furthermore, between 10 minutes and 3 hrs of TQ treatment, smear-like bands of UHRF1 appeared with MW between 115 and 250 kDa (Figure 3A). Of note, the down-regulation of HAUSP expression, perfectly paralleled the appearance of the higher bands of UHRF1 suggesting a strong correlation between these two events. In contrast, p73 and cleaved caspase-3 began to appear, not when higher bands of UHRF1 appeared, but only when UHRF1 began to decrease, *i.e.,* after 1 hr (Figure 3A), showing that cleaved caspase-3 and p73 induction are related to UHRF1 down-regulation.

The occurrence of smear-like bands above the usual expected MW of a protein is a hallmark of post-translational modifications such as ubiquitination or SUMOylation. To investigate whether TQ induces UHRF1 ubiquitination, Jurkat cells were exposed to 30 µM of TQ for 10 minutes and then the ubiquitination of UHRF1 was studied by co-immunoprecipitation experiments. Interestingly, when we immunoprecipitated UHRF1 using anti-UHRF1 antibody, we found that UHRF1 was ubiquitinated in TQ-treated Jurkat cells (revealed with an anti-ubiquitin antibody), while such effect was not observed in TQ-untreated Jurkat cells (Figure 3B). These findings indicate that TQ induces a fast ubiquitination of UHRF1which could be a key event that determine the onset of its degradation and subsequent activation of apoptosis.

### Analysis of UHRF1 complex content in terms of E3 ligases and deubiquitinases

To disentangle the mechanism underlying the ubiquitination of UHRF1, we aimed to purify the UHRF1 complex in HeLa cells treated or not with TQ. We switched to HeLa cells as they represent a well-accepted cancer model and constitute a much more convenient cell line for establishing a stable cell line expressing tagged proteins [40, 41]. Nevertheless, HeLa cells are less sensitive to TQ compared to Jurkat cells [29, 42] and this is why we used higher TQ concentrations. We found that several E3 ligases appeared in the UHRF1 complex, while several deubiquitinases moved from the complex upon TQ treatment (Table 1). However, we did not observe the presence of SCF (β-TrCP) that has been identified as being an E3 ligase that ubiquitinates UHRF1 upon DNA damage [43]. In contrast, we found three E3 ligases that were significantly enriched in UHRF1 complexes upon treatment with TQ (Table 1). These E3 ligases were UBR5 (Ubiquitin Protein Ligase E3 Component N-Recognin 5), DDB1-CUL4A and HUWE1. Two other E3 ligases, namely UBR4 and RING1, were found but only in the UHRF1 complex purified from TQ-treated cells and in a weak amount (Table 1). UBR5 contributes to tumor initiation and progression [44]. CUL4A-DDB1 tandem functions as an oncogene [45, 46], HUWE1 has rather a tumor suppressor activity [47, 48]. In parallel, we observed the presence of several deubiquitinases, among which the major was USP7 confirming previous reports that this is the major deubiquitinase regulating UHRF1 stability [38, 39, 49, 50]. Considering that several E3 ligases have been identified, we first intended to determine the putative contribution of the auto-ubiquitination activity of UHRF1.

**Table 1:** Mass spectrometry data of E3 ligase and deubiquitinase found in the soluble nuclear extract of epitope tagged UHRF1 in the absence or presence of 100 µM of TQ

		Peptide	Peptide	Enzyme
Entry	Protein	Control	TQ-treated	
Q96T88_UHRF1_HUMAN	UHRF1	77	68	E3-ligase
O95071_UBR5_HUMAN	UBR5	0	45	E3-ligase
Q16531_DDB1_HUMAN	DDB1	10	20	E3-ligase
Q13619_CUL4A_HUMAN	CUL4A	2	5	E3-ligase
Q7Z6Z7_HUWE1_HUMAN	HUWE1	2	63	E3-ligase
Q5T4S7_UBR4_HUMAN	UBR4	0	1	E3-ligase
Q06587_RING1_HUMAN	RING1	0	1	E3-ligase
Q93009_UBP7_HUMAN	USP7	46	32	deubiquitinase
P45974_UBP5_HUMAN	USP5	9	0	deubiquitinase
Q86UV5_UBP48_HUMAN	USP48	5	0	deubiquitinase
Q9Y4E8_UBP15_HUMAN	USP15	4	0	deubiquitinase
Q53GS9_SNUT2_HUMAN	USP39	3	0	deubiquitinase
Q93008_USP9X_HUMAN	USP9X	3	0	deubiquitinase

### Disturbing the RING domain of UHRF1 abolishes TQ-induced UHRF1 ubiquitination

In order to investigate, the contribution of the UHRF1-RING domain in its ubiquitination, we constructed cell lines stably expressing HA-tagged UHRF1 bearing, or not, a point mutation in the RING finger (C724A, Supplementary Figure 1A) thus abolishing endogenous E3 ligase activity [51]. HeLa cell lines adequately express wild type HA-tagged UHRF1 and mutated HA-tagged UHRF1 as analyzed by western blot (Supplementary Figure 1B). Immunocytochemistry experiments were carried out to control whether localization is limited to the nucleus for both cell lines, *i.e.,* mutated and wild-type (Supplementary Figure 1C). The obtained data showed that mutated UHRF1 remained always in the nucleus and the mutation did not induce mislocalization (Supplementary Figure 1C).

**Figure 4:**
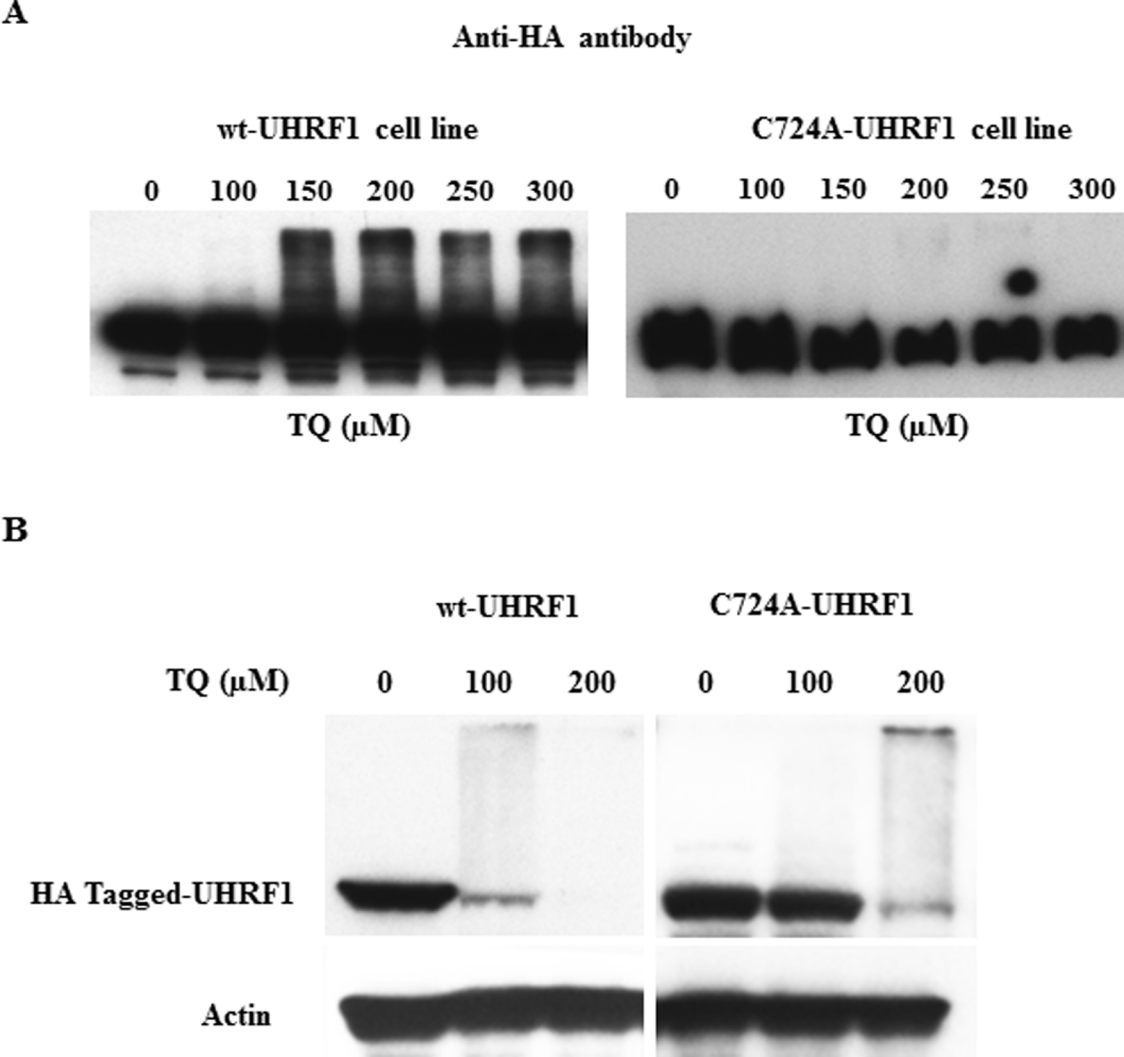
Effect of TQ on UHRF1 expression in HA-tagged UHRF1 wild-type and HA-tagged RING-mutated UHRF1 cell lines HeLa cells were treated with various concentrations of TQ for 3 hrs (**A**) and for 24 hrs (**B**) and the expression of HA-tagged UHRF1 was investigated using a monoclonal anti-HA antibody. Data are representative of 2 different experiments.

We then studied the effect of TQ on UHRF1 ubiquitination in both cell lines, *i.e.,* mutated or not in the RING domain of UHRF1. Between 150 µM and 300 µM, TQ induced a dose-dependent ubiquitination of HA-tagged UHRF1, after 1 hr treatment. In contrast, ubiquitination was not observed in the RING-mutated HA-tagged UHRF1 for the same concentration of TQ (Figure 4A). After a 24 hrs treatment, we could observe that a TQ concentration of 100 µM, only for the wild-type HA-tagged UHRF1 was ubiquitinated whereas the RING mutated HA-tagged UHRF1 was not (Figure 4B). However, as soon as ubiquitination occurs, (at 200 µM of TQ) degradation of UHRF1 is promoted (Figure 4B). These findings indicate that through its RING domain, UHRF1 commits an auto-ubiquitination in response to TQ, as such effect was not observed in the cells expressing the mutant RING domain.

### The role of auto-ubiquitination of UHRF1 challenged by TQ

**Figure 5:**
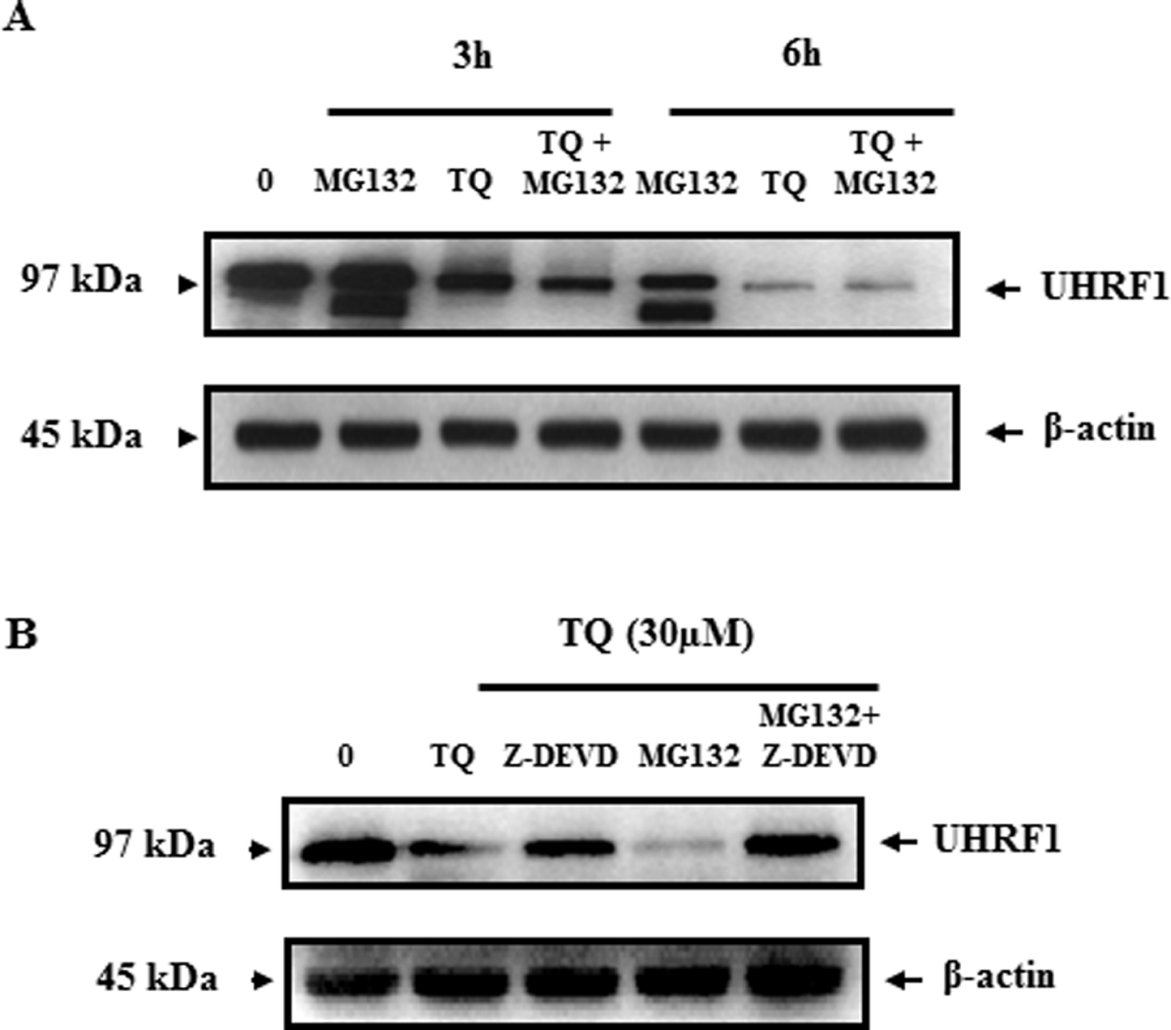
Effect of TQ on UHRF1 expression in the presence of the proteasome inhibitor MG132 and the caspase-3 inhibitor Z-DEVD (**A**) Jurkat cells were pretreated with proteasome inhibitor MG132 for 1 h before adding TQ for 3 and 6 h. (**B**) Jurkat cells were pretreated with either caspase-3 inhibitor (Z-DEVD) at 3 µM or proteasome inhibitor MG132 at 10 µM or both inhibitors for 1 h before adding TQ for 24 h. Western blot was then performed using anti-UHRF1 antibody. Data are representative of 3 different experiments.

Further investigations were undertaken to understand the consequences of auto-ubiquitination of UHRF1. Consistent with the assumption that polyubiquitination occurs in order to conduct targeted proteins to proteasome-dependent degradation, we studied the effect of MG132, an inhibitor of the proteasome, on the expression of UHRF1. We found that treating the cells with MG132 had no effect on the expression of UHRF1 neither after 3 hr, 6 hr (Figure 5A) nor 24 hr (Figure 5B). Interestingly, in the presence of Z-DEVD, an inhibitor of caspase-3, expression of UHRF1 could be recovered or maintained (Figure 5B). This result is in accordance with the data obtained in Figure 2A, in which UHRF1 is disappearing as soon as caspase-3 is appearing. This correlation supports a strong relationship between these two events. Altogether, these results suggest that caspase-3 is involved in the degradation of ubiquitinated UHRF1.

## DISCUSSION

UHRF1 plays a fundamental role in silencing of TSGs. Interestingly, always when UHRF1 is down-regulated through the use of natural anti-cancer compounds, TSGs are expressed again with subsequent induction of apoptosis [7, 8]. Therefore, it is of high interest to decipher the mechanism of UHRF1 down-regulation putatively highlighting new strategies of TSGs re-expression and thus of anti-cancer therapies. We observed that TQ induced a fast and abundant polyubiquitination of UHRF1 leading to nearly a tripling of its MW, suggesting that about 20 molecules of ubiquitin have been attached to UHRF1. To our knowledge, it is the first time that polyubiquitination of UHRF1 has been visualized *in vivo* in cells, upon treatment with a natural compound or by physical DNA damage even when an E3 ligase, other than UHRF1, is involved in the proteasomal degradation [43]. Interestingly, the transcription of *UHRF1* was stopped by TQ, which prevents compensation of its degradation.

By analyzing the UHRF1 complex, we found a network of E3 ligases and deubiquitinases (Table 1) suggesting that UHRF1 may play a central role in ubiquitin-mediated regulation of gene silencing. HAUSP was found to be the most abundant deubiquitinase, in accordance with previous reports highlighting this enzyme as a protector of UHRF1 [38, 49, 50]. Several E3-ligases were found in the UHRF1 complex, some of which yielding peptides in high amount, comparable to that of UHRF1. These E3 ligases are UBR5 and HUWE1, which, notably appeared to be increased upon exposure of cells to TQ (Table 1). Although, such behavior would support a targeting of UHRF1 by these E3 ligases, our results demonstrated that TQ induces an auto-ubiquitination rather than the intervention of other E3 ligases. However, we do not exclude that possibility since a slight ubiquitination persists when the RING domain was mutated (see Figure 4A, at 250 µM and 300 µM of TQ). However, this weak ubiquitination may also come from either a remaining ligase activity of UHRF1 as the mutation might not completely abolish its intrinsic E3 ligase activity or from one of the E3 ligases present in the UHRF1 complex, such as UBR5 or HUWE1 or CUL4A/DDB1 (Table 1). We did not observe the presence of SCF (β-TrCP), the sole E3 ligase reported so far to ubiquitinate UHRF1 and to further challenge its degradation via a proteasome-dependent process [43]. If there is an intervention of another E3 ligase, such as one of those mentioned above, we suggest that it will enter into play in a time-dependent manner after the auto-ubiquitination process. Our results clearly show a strong correlation between the TQ-induced ubiquitination of UHRF1 and its degradation.

We observed that degradation of UHRF1 is not going through the proteasome as it was shown for UV-radiation [43] since MG132 was inefficient in recovering UHRF1 expression. This is not absolutely surprising considering that TQ has been reported to show proteasome inhibitory capacity [52]. In contrast, UHRF1 down-regulation induced by TQ could be countered when we used the caspase-3 inhibitor, Z-DEVD. We have no explanation for the discrepancy between our study and the previous report [43] but it might arise from different pathways. One interesting hypothesis is that ubiquitination of UHRF1 by SCF (β-TrCP) following UV-induced DNA damage, drives degradation via the proteasome whereas auto-ubiquitination of UHRF1 drives degradation via activation of caspase-3. Accordingly, it has recently been shown that UHRF1 down-regulation in cancer cells induced caspase-8 dependent apoptosis and the activation of caspase-3 [53]. We suggest, that auto-ubiquitination inactivates UHRF1, mimicking a down-regulation, and by this way activates caspase-3, which further activates its degradation. Consistently, with this, polyubiquitination does not always associate with proteosomal degradation. as the activity of the transcription factor ReIB was shown to be enhanced by polyubiquitination [54].

Autoubiquitination and ubiquitination of target proteins are described as the general function of most proteins containing the RING domain [55–57]. There is a plethora of targets of UHRF1, such as HSP90, DNMT3b [58–60], Trim28, H3K18 and PCNA-associated factor 15 [34, 59, 61, 62]. Therefore, we do not exclude that polyubiquitination enhances UHRF1 E3 ligase activity, in order to fulfil its role in participating in DNA repair process [63] before its degradation by caspase-3. This would be in accordance with the ability of TQ to cause DNA damages in Jurkat cells [29].

In conclusion, the present study shows that TQ induces, through its RING domain, a rapid UHRF1 poly-auto-ubiquitination, leading to apoptosis. The TQ-induced UHRF1 ubiquitination could be a result of HAUSP down-regulation. However, TQ-induced HAUSP/UHRF1 deregulation needs further investigation to decipher the molecular events involved, namely the HAUSP down-regulation, which triggers UHRF1 down-regulation followed by apoptosis. Our study is further in accordance with reports from other laboratories supporting HAUSP as an interesting anti-cancer therapy [64–66]. Our study also provides new insights into the regulation of UHRF1 expression upon treatment with natural anti-cancer drugs.

## MATERIALS AND METHODS

### Cell culture and treatment

Human T lymphocyte cell line Jurkat, HL60 and HeLa cell lines were obtained from the America Type Culture Collection (Mannassa, VA, USA). Cell lines were maintained in a humidified incubator with 5% CO2 at 37° C, and were grown in RPMI 1640 (Sigma-Aldrich, St-Louis, MO) for Jurkat cells and HL60 cells and EMEM (Biowhitaker, Lonza, Belgium) for HeLA cells. All media were supplemented with 15% (v/v) fetal calf serum (FCS, Biowhitaker, Lonza, Belgium), 2 mM glutamine, 100 U/ml penicillin and 50 μg/ml streptomycin (Sigma St. Louis, MO). For all treatments, a 100 mM solution of TQ (Sigma–Aldrich, Louis, MO, USA) was prepared in 100% DMSO (DiMethylSulfOxide; Millipore, Molsheim, France) and appropriate working concentrations were prepared with cell culture medium. The final concentration of DMSO was always less than 0.1% in both control and treated conditions. Proteasome inhibitor, MG132, and Caspase-3 inhibitor, ZEDV, were obtained from Sigma-Aldrich and from Gentaur Europe (Kampenhout, Belgium).

### Immunofluorescence

Regarding that UHRF1 is HA-tagged in these cells, immunofluorescence was performed using rat anti-HA antibody (Roche Diagnostics, Mannheim, Germany) following standard procedures. Anti-HA antibody was used at 1/200 dilution, the secondary antibody used is a goat anti-rat IgG coupled to Alexa Fluor 488 (Molecular Probes) at a dilution of 1/400.

### Cell lines and complexes purification

The coding sequence of UHRF1 was mutated using megaprimer PCR procedure to produce C724A mutant protein (Figure 2A). This strategy has been shown to disrupt the RING domain of UHRF1 [51]. The complete coding sequence (WT and C724A) of UHRF1 was subcloned into the XhoI-NotI sites of the pOZ-N retroviral vector to produce UHRF1 protein fused with N-terminal Flag- and HA-epitope tags (e-UHRF1). e-UHRF1 was stably expressed in HeLa cells by retroviral transduction as described elsewhere [67]. e-UHRF1 nuclear complex (e-UHRF1.com) was purified from these cells by double immunoaffinity as described hereafter.

### Double-immunoaffinity purification

Cells were lysed in hypotonic buffer (10 mm Tris-HCl at pH 7.65, 1.5 mm MgCl2, 10 mm KCl) and disrupted by Dounce homogenizer. This extract was then centrifuged at 4° C to separate the cytosolic fraction from the pellet. The nuclear-soluble fraction was obtained by incubation of the pellet in high-salt buffer (to get a final NaCl concentration of 300 mM). Tagged UHRF1 was then immunoprecipitated with anti-Flag M2-agarose (Sigma), eluted with Flag peptide (0.5 mg/mL), further affinity-purified with anti-HA antibody-conjugated agarose (Sigma), and eluted with HA peptide (1 mg/mL). The HA and Flag peptides were first buffered with 50 mM Tris-Cl (pH 8.5), then diluted to 4 mg/mL in TGEN 150 buffer (20 mM Tris at pH 7.65, 150 mM NaCl, 3 mM MgCl2, 0.1 mM EDTA, 10% glycerol, 0 0.01% NP40), and stored at −20° C until use. Between each step, beads were washed in TGEN 150 buffer. Complexes were resolved by SDS-PAGE and stained using the Silver Quest kit (Invitrogen).

### Western blot analysis

The cells were treated with different concentrations of TQ for different times. The cells were then harvested, centrifuged to discard the RPMI medium, washed with cold PBS (phosphate buffered saline), resuspended in RIPA buffer (25 mM Tris, pH 7.6, 150 mM NaCl, 1% NP-40, 1% sodium deoxycholate and 0.1% SDS; Sigma–Aldrich, USA) containing protease inhibitors. Equal amounts of total protein were separated on 10–12% polyacrylamide gel and electrophoretically transferred to a nitrocellulose membrane. After blocking with 5% non-fat dry milk or 3% BSA (Bovine Serum Albumin) and tween 20 in PBS, the nitrocellulose membranes were incubated, at 4° C overnight, with either mouse monoclonal anti-UHRF1 antibody [68], mouse monoclonal anti-HAUSP antibody (Santa Cruz biotechnology), mouse monoclonal anti-p73 antibody (BD Biosciences, Pharmingen), a mouse monoclonal anti-ubiquitin (Santa Cruz biotechnology), rabbit polyclonal anti cleaved caspase-3 antibody (Cell Signaling Technology, Danvers, MA, USA), monoclonal anti-HA antibody 9E (Roche Diagnostics) or mouse monoclonal anti β-actin antibody (Abcam, Paris, France), according to the manufacturer's instructions. The membranes were then washed three times with PBS for 10 min. Membranes were, thereafter, incubated with the appropriate horseradish peroxidase-conjugated secondary antibody (diluted to 1:10,000 for anti-mouse anti-bodies and 2: 10,000 for anti-rabbit antibody) at room temperature for 90 min. The membranes were then washed with PBS five times. Signals were detected by chemiluminescence using the ECL Plus detection system (Amersham, GE Healthcare UK). For UHRF1 protein quantification, images of Western blots were processed using NIH ImageJ software.

### Immunoprecipitation assays

Cells were treated with 30 µM of TQ for 10 minutes, washed with cold PBS and then the proteins were extracted as described above. First, 2 µg of a specific monoclonal anti-UHRF1 antibody was incubated for two hours at 4° C with protein G-sepharose beads (Amersham Bioscience Limited) in PBS supplemented with a cocktail of protease inhibitors (Roche Diagnostics). The mixture was then centrifuged at 4,500 rpm for 3 minutes at 4° C and washed three times in the same conditions. Second, 1 mg of Jurkat protein lysates was incubated overnight at 4° C with the protein G-sepharose beads coupled with the anti-UHRF1 antibody. Finally, beads were washed five times in 1 mL of PBS and bound proteins were removed from the beads and denatured by Laemmli solution containing 5% mercaptoethanol and separated on SDS–PAGE as described above. Then, Western-blot was performed by using a specific mouse monoclonal anti-UHRF1 antibody [68] and a specific mouse monoclonal anti-ubiquitin antibody (Abcam, Paris, France).

### Apoptosis assays

Cell apoptosis rate was assessed by flow cytometer (BD FACS Calibur system, BD Biosciences, San Diego, CA, USA) using the Annexin V-FITC/propidium iodide (PI) apoptosis assay (BD Biosciences), following the manufacturer's recommendations. CellQuest software (BD Biosciences) was used for the analysis of the data.

### Real-time RT–PCR analysis

Real-time RT-PCR analysis was described elsewhere [22]. Briefly, Jurkat cells were treated with TQ for 3 and 6 hours, then total RNAs were purified and subjected to reverse transcription using Oligo(dt) (Sigma) and Superscript II reverse transcriptase (Invitrogen). Quantitative real-time PCR was done with the LightCycler 480 SYBR Green I Master kit (Roche Diagnostics) and the Mastercycler Realplex apparatus (Eppendorf, Montesson, France). The results were normalized with GAPDH mRNA. The sequences of the primers for PCR amplification were: UHRF1 (sense: 5′-GTCGAATCATCTTCGTGGAC-3′; antisense: 5′-AGTACCACCTCGCTGGCAT-3′); GAPDH (sense: 5′- GGTGAAGGTCGGA-GTCAAC-3′, antisense: 5′-AGAGTTAAAAGC-AGCCCTGGTG-3′); p73 (sense: 5′- ACAGCACCTACTTCGACCTT-3′, antisense: 5′- CCGCCCACCACCTCATT-3′). Amplicons were size controlled on agarose gel and purity was assessed by analysis of the melting curves at the end of the real-time PCR reaction.

### Statistical analysis

Results are presented as mean ± SEM of at least three independent experiments. Statistical analysis was performed using a two-way analysis of variance (ANOVA) followed by a Bonferroni post-hoc test to compare differences. Significant differences are indicated as ^*^P < 0.05, ^**^P < 0.01, ^***^P < 0.001, ^****^P < 0.0001.

## SUPPLEMENTARY MATERIALS FIGURE



## Abbreviations

UHRF1: Ubiquitin-like containing PHD and RING Finger domains 1
TSGs: Tumor suppressor genes
HDAC1: Histone deacetylase 1
DNMT1: DNA methyltransferase 1
Tip60: Tat interactive protein
HAUSP: Herpes virus-Associated Ubiquitin-Specific Protease
RING: Really Interesting New Gene domain
ECREM: Epigenetic Code Replication Machinery
TQ: Thymoquinone
UBR5: Ubiquitin Protein Ligase E3 Component N-Recognin 5
