# Supplementary material for: Thymoquinone challenges UHRF1 to commit auto-ubiquitination: a key event for apoptosis induction in cancer cells
Source: Oncotarget. 2018 Jun 19;9(47):28599–611. doi: 10.18632/oncotarget.25583 (PMC6033341; doi:10.18632/oncotarget.25583)
Supplement: Supplementary file 1 [file oncotarget-09-28599-s001.pdf]

Thymoquinone challenges UHRF1 to commit auto-ubiquitination: a key event for apoptosis induction in cancer cells

SUPPLEMENTARY MATERIALS

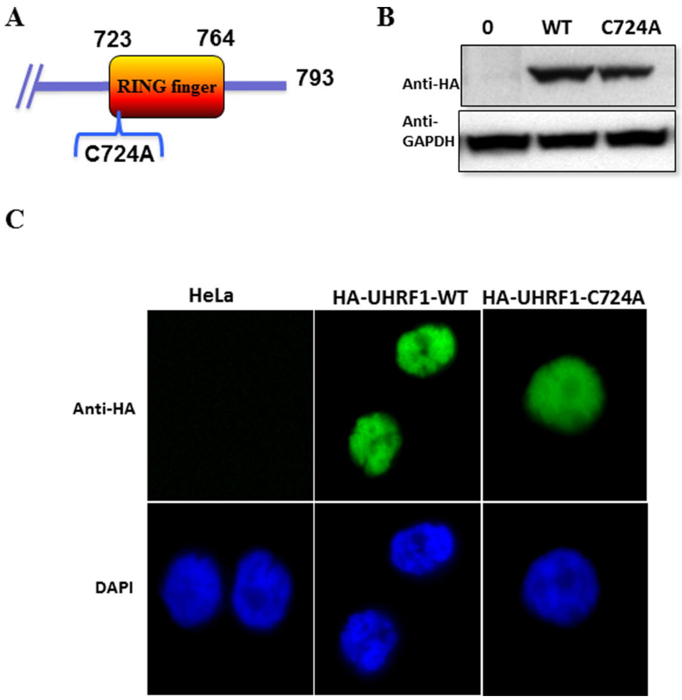

Supplementary Figure 1: Construction of a UHRF1 RING mutated stable HeLa cell line. (**A**) Localization of the mutation of the RING domain of UHRF1. Cysteine 724 was substituted by an alanine disrupting the zinc finger structure of this domain. (**B**) Western blot of HeLa cell extracts to confirm that cell lines contain HA-tagged UHRF1. An anti-HA monoclonal antibody was used to reveal HA-tagged UHRF1. (**C**) Immunocytochemistry of HeLa cell lines containing wild-type (WT) or mutated UHRF1 (HA-UHRF1-C724A) to investigate sub-cellular localization. The same antibody as for Western blot was used. A secondary goat anti-rat coupled to Alexa Fluor 488 was used.
